# Drought tolerance induction and growth promotion by indole acetic acid producing Pseudomonas *aeruginosa* in *Vigna radiata*

**DOI:** 10.1371/journal.pone.0262932

**Published:** 2022-02-04

**Authors:** Malika Uzma, Atia Iqbal, Shahida Hasnain

**Affiliations:** 1 Department of Microbiology and Molecular Genetics, The Women University, Multan, Pakistan; 2 Department of Microbiology and Molecular Genetics, University of the Punjab, Lahore, Pakistan; University of Agriculture, PAKISTAN

## Abstract

Drought accompanied with reduced precipitation is one of the key manacles to global agricultural throughput and is expected to escalate further hence posing major challenges to future food safety. For a sustainable agricultural environment, drought resistant plant growth promoting rhizobacteria (PGPR) are new encouraging prospect, which are inexpensive and have no side effects, as those of synthetic fertilizers. In the present study, five strains of *Pseudomonas aeruginosa*, the strain MK513745, strain MK513746, strain MK513747, strain MK513748, and strain MK513749 were used as drought tolerant PGPR with multiple traits of IAA production, N fixation, P solubilization, siderophore producing capabilities. The strain MK513745 and strain MK513749 produced higher quantities of indole acetic acid (116±0.13 and 108±0.26 μg ml^-1^). MK513749 yielded 12 different indole compounds in GCMS analysis. The strain MK513748 yielded maximum S.I. (3.33mm) for phosphate solubilizing test. Maximum nitrogen concentration was produced (0.18 μg ml^-1^) by strain MK513746. Percent siderophore units ranged from 2.65% to 2.83% as all five pseudomonas strains were siderophore positive. In all growth experiments of plant microbe interaction two varieties of *Vigna radiata* (AZRI-06, NM-11) plants inoculated with *P*. *aeruginosa* strains under drought stress responded significantly (P**<**0.05) better than control stressed plants. Maximum shoot length was enhanced up-to 125%, pod/plant 172%, number of grains 65%, 100 seed weight 95%, 100 seed straw weight 124% and total yield 293% were recorded in plants inoculated with drought stress tolerant PGPR in both varieties as compared to respective stressed control plants. Photosynthetic activity, membrane stability (45%), water content (68%) and antioxidant efficacy (19%) were improved with PGPR inoculations. The variety NM-11 (V2) was more tolerant to drought stress with inoculations of Pseudomonas strains than AZRI-06 (V1). Inoculations with these indole acetic acid producing strains would be suitable for plant growth promotion in areas facing water deficiency.

## Introduction

Water stress is a serious problem in plant production as it imparts drastic consequences at all stages [1,2]. Up to 45% of the world’s agricultural lands are subjected to continuous or frequent drought wherein 38% of the world human population resides [3]. This problem is since decades due to global warming, low rainfall, high temperature and reduced reservoir capacity. The agricultural regions that are affected by drought can experience yield loss up to 50% or more [4,5]. According to the United Nations Population Fund (UNFPA) global population will might reach 11.2 billion by 2100 [6,7]. Pakistan is also an agriculture-based economy and agriculture is the prime sector to be affected by the increased unavailability of fresh water [8]. Pakistan is 6th most drought prone country of the world and more than 2.5 million people are affected by drought only in Sindh. In Pakistan, nearly 15 million hectares of cultivated land are affected by aforementioned conditions resulting in reduced productivity and the situation can be alarming by the year 2025 [9,10].

Mung bean (*Vigna radiata* L.) is one of the important conventional pulse crops of Pakistan and commonly known as green gram. Globally, 90% of its production is from Asia and Pakistan is amongst the key producers along with India, China and Thailand [11]. It has high nutritive value, and due to this, has advantage over the other pulses. The seed contains 24.20% protein content, 1.30% fat, and 60.40% carbohydrates and contains appreciable quantities of calcium and phosphorus 118 and 340 mg/100 g of seed, respectively [12]. Moreover, mung bean has lower percentage of phytic acid (72% of total phosphorus content) that is mostly found in leguminous and cereal crops and is reported to have a negative impact on bioavailability of iron and zinc in plant-based foods. In baby foods, mung bean is being used due to its nutritional value, better taste and an iron-rich source [13]. It is also grown during spring season mainly in southern Punjab and Sindh province. Area under cultivation of mung bean in Pakistan is 130.90 thousand hectares [14]. Punjab is the major mung bean growing province that alone accounts for 88% area and 85% of the total mung bean production. The average yield of mung bean in Pakistan is 461.50 kg ha^−1^ and its seed production is 92.90 thousand tones, which is much lower than the harvested potential of our existing varieties. The decreased production of mung bean is due to abiotic and biotic stresses, low quality seeds and poor crop management practices of farmers [15,16] Due to static production of mung bean during the last decade, the gap between supply and demand has become wider [12]. Therefore, it is necessary to advance the weak chains of this crop production.

The improvement of soil fertility is one of the strategies commonly used to increase agricultural production and PGPR participate in soil fertilization through the bio fixation and bio-solubilization process. The beneficial microbiome associated with roots and plant tissues alleviate plant stress by a variety of mechanisms [17,18]. Generally, IAA released by rhizobacteria interferes with many plant developmental processes because the endogenous pool of plant IAA may be altered by the acquisition of IAA that has been secreted by soil bacteria. It is synthesized by several independent biosynthetic pathways and mostly produced in bud and young leaves of plant. In young stems IAA causes a rapid increase in cell wall extensibility and promotes growth of auxiliary bud and bud formation. Moreover, it helps in the apical dominance in plants, and also stimulates lateral and adventitious root development and growth [19]. Besides development, IAA plays crucial role in leaf and flower abscission as well and can be considered as the major auxin involved because it plays overall role in growth stimulation by being involved in DNA synthesis [20].

Auxin producing bacteria are reported to enhance root lengths, their number of tips, surface area and plant biomass [21] moreover they play important role in growth regulation aiding in expansion of cotyledons, root hair formation during seed germination [22]. In plant microbe-interactions indole acetic acid signalling is involved in lateral root proliferation and stress tolerance induction. Plant growth promoting bacteria also up-regulate genes expressions for nitrogenase activity in host plants and relieve from different abiotic stresses [23,24]. Moreover, IAA producing PGPRs are reported to boost nutrient uptake, chlorophyll contents and upregulate genes involved in transportation of heavy metals [25] either directly increasing micronutrient uptake and influencing phytohormone homeostasis, or indirectly by stimulating the plant immune system against phytopathogens and stresses [26]. Furthermore, Bacterial IAA stimulates the synthesis of 1-aminocyclopropane-1-carboxylate (ACC) deaminase, which destroys an ethylene precursor ACC, a stress hormone hence delaying senescence in plants under stress [27]. Agriculture is considered as most vulnerable sectors to climate-change. Exploiting plant-microbe interaction is a relevant approach to increase food production for the growing population in the current scenario of climate change. The objective of current study was to isolate indole acetic acid producing rhizobacteria and evaluation of their unique PGP traits, like nitrogen fixation, phosphorus solubilization, siderophore, ACC deaminase, other hydrolytic enzyme producing capabilities and drought tolerance (up-to 20% PEG stress). Selection and application of suitable strains on growth promotion of *Vigna radiata* under drought stress in lab and field conditions.

## Materials and methods

### Isolation, purification, characterization of rhizobacteria

A variety of rhizospheric soil samples from various plants were collected at geographical co-ordinates 31°N and 72°E to isolate IAA producing rhizobacteria. Soil samples and adhering roots were collected, packed in polyethylene bags, labelled, and stored at 4°C immediately [28]. One gram of each soil sample was used for serial dilutions using autoclaved water up to10^-6^ by using serial dilution method [29]. A 0.1 ml of each dilution (10^−1^ to 10^−6^) was inoculated into autoclaved LB broth and incubated in an incubator shaker at 25±2°C for 24 hours. Each incubated dilution was spread on agar plates. After a 24-hour incubation period, colonies were observed and selected for isolation. Characterization of isolates was done following the Bergey’s Manual for bacterial systematics [30].

### Drought tolerance assay

Screening for drought tolerance of isolates was done by using different levels of polyethylene glycol (PEG) 6000 (5%, 10%, 15%, and 20% by dissolving 5, 10, 15 and 20 g of PEG in 100 ml autoclaved distilled water respectively) following Busse and Bottomley’s standard protocol [31]. Broth with developed PEG levels was inoculated with selected isolates and incubated in an incubator shaker (Robus technology, S1900R) at 25±2˚C for 36 hours. After incubation the broth cultures were centrifuged and supernatants were used to check optical densities.

### Evaluation of PGP traits

Screening for IAA production was done by using Salkowsky’s reagent [32] and concentrations of IAA were determined using a standard curve prepared from pure IAA solutions [33]. Thin layer chromatography [34] was performed with ethanolic extracts of bacterial secondary metabolites. Strains were further processed for IAA quantification. Ethyl acetate extracts in ethanol were used for GCMS (gas chromatography mass spectrometry, GCMSqp 5050 shimadzu japan) by following the protocol of Tien et al [34].

Isolated bacteria were also checked for their phosphate solubilizing efficiency using NBRIP medium containing insoluble phosphate in form of tricalcium phosphate [35]. Bacterial cultures were further grown in triplicate using Pikovskaya broth at 30±2˚C at 150 rpm in an incubator shaker. After centrifugation (Herolab Ludwig- wagner-str) at 4000 rpm for 10 minutes at 4°C, phospho molybdate blue color method [36] was adapted to quantify phosphorus solubilization. Optical densities were recorded at 351 nm by spectrophotometer (S-200D R & M) and calibration was done by KH_2_PO_4_.

Isolated strains were also screened for N fixing capabilities. Isolates were measured for their selective aptitudes index values created on calculation of fraction of total diameter and colony diameter (Total diameter = colony diameter + hallow zone diameter). Strains possessing abilities to fix nitrogen were quantified using Nessler’s reagent. For standard curve ammonium sulphate concentrations were used as reference [37].

Pure colonies of isolated bacteria were screened for siderophore production [38] by chrome azeurol test and Quantitative estimation was done by siderophore shuttle assay using MEB (malt extract broth) [39]. Percent siderophore units were calculated by dividing the difference in absorbances (of reference and sample) with absorbance of reference and then multiplying with 100 (% units of siderophore = Ar-As/ Ar x 100).

Selected isolates were checked for their efficacy to produce hydrogen cyanide (HCN). Nutrient broth supplemented with 4.4 g/l of glycine was used for modified agar. The media plates were poured, spot inoculated. Whatman filter papers dipped in solution of 2% sodium carbonate and 0.5% picric acid were placed in lids of each Petri plate. The plates were incubated (inverted position) for 48–96 hours. After incubation plates were observed for color changes from yellow to orange brown [40].

ACC deaminase production activity was also checked for each selected strain. Dworkin and Foster (DF) medium was prepared to check this activity [41]. Media plates were inoculated respectively and incubated for 3–7 days. Production of white zones was a positive indication of ACC deaminase production.

### Phylogenetic identification of rhizobacteria

Fresh cultures (24 hours incubated) of drought tolerant rhizobacteria were ship to Macrogen Inc. (Korea) for ribotyping. Sequences obtained from Macrogen were analyzed through Blast (NCBI) and comparison was made with online database. The evolutionary history of rhizobacteria was determined by N.J (neighbor-joining) method of [42]. Molecular evolutionary genetic analysis was done by using software MEGA 7 to construct optimal phylogenetic tree [43].

### Plant microbe interactions

#### Layout, treatments and design

Experiments were conducted under axenic and natural conditions in 2017–2020 in late summer (Kharif) season (July-October) in Multan (Geographical coordinate: 30.16° N, 71.52° E). The seeds of two varieties of *Vigna radiata* (V1: AZRI 2006 and V2: NM-2011) were obtained from Punjab seed corporation, Multan. The experimental station is Department of Microbiology and Molecular Genetics, The Women University, Multan, Pakistan. In all experiments, five PGPR strains were inoculated along with two controls (water and stressed) and the total thirteen treatment combinations were laid out in a Randomized Block Design with three replications.

### Growth promotion of *Vigna radiata*

Selected Strains were used for drought alleviation of mung bean (both varieties) in Lab, pot and field trials. In lab trials axenic conditions (Autoclaved soil, temperature 25±1°C, 12 hours photoperiod with light intensity of 2200 lux regime) were maintained. Four experimental sets of seeds control watered (C.W), control stressed (C.S) without inoculations and inoculated with PGPR at PEG 10% (10 g in 100 ml of distilled water), PEG 20% (20 g in 100 ml of distilled water) irrigation levels were used. Control watered (C.W) treatments received same amount of autoclaved distilled water without PEG. There were three replicates for each treatment. Total 78 plastic cups (3.6″×2.7″) with RCBD and two factor factorial arrangements were used in growth room for both varieties. Mung bean seeds were surface sterilized with Na-hypochlorite (1%) for 20 minutes and then rinsed with distilled water and dipped in 10 ml culture of selected strains and 0.1% carboxy methyl cellulose (CMC) as adhesive for half an hour to prepare for inoculation. Fresh bacterial cultures (24 hours incubated) were used for this purpose. Different growth parameters were checked after fourteen days. Each plastic pot contained three replicates and there was replication of three pots for each treatment.

Plant microbe interactions were checked under natural conditions after lab environment. Pot experiment was conducted in 48 earthen pots (12.7″×12″) at 75% and 50% field capacity with inoculated plants. Oven dried half kilo gram of soil was reweighed. Water was added to that soil and paste was formed to get its saturation point with regular stirring. The point at which water accumulated on surface was considered to be its saturation point. Half to this was taken its 100% field capacity and accordingly 75%, 50% water capacity of half kg soil [44]. There were two types of control plants un-inoculated watered (C.W), un-inoculated stressed (C.S) plants. Stress was induced after 3 weeks of germination [45]. Inoculations were done as (10^6^ cfu/ml). Three replicate pots for each treatment were placed in complete randomized block design. The response of plants in the field may entirely be different from lab experiments or in natural environment at limited scale (pot experiment), hence field trials were accomplished. For field experiments, treatments applied were control un-inoculated with water and stressed treatments along with inoculated stressed plants. There was split plot arrangement. Each plot was 2´×5´ long with 30cm row- row spacing. The spacing between every plant was kept 20 cm. the seeds of both varieties were surface sterilized and dipped in inoculums as above in pre-labelled petri dishes for half an hour before sowing. The plants were exposed to 2, 4 and 8 days of drought stress [45] and recovery. After one week of germination, seedlings were again supplemented with broth inoculums by syringe method. The inoculations were done as number of bacteria was 10^6^ cfu/plant. Un-inoculated plants received same amount of growth medium without bacteria. Plants were irrigated until 30 days, then subjected to drought stress (5–7 days) and watered again for recovery (for 3 days). Plants grown in all trials were measured in terms of root length (cm), shoot length (cm), root fresh weight, (gm), shoot fresh weight (gm) and plant dry biomass (gm). A 0.1 g fresh leaf incubated with acetone was centrifuged and absorbance of supernatant of each treatment was recorded at 645 and 663 nm to calculate the chlorophyll pigments [46]. Relative electrolyte leakage (REL) of 0.5 g fresh leaves discs, was calculated by finding electrical conductivities (C1 and C2) by EC meter at 40°C and 100°C respectively. Percentage of relative water content (RWC) was estimated by using fresh plant leaves immersed in deionized water and taking difference in weights before and after immersion as described by Flower and Ludlow [47]. Stress enzyme and proline activity in plants under drought stress was determined by Bates’s method [48]. For antioxidant enzyme analyses 0.5 g fresh leaves of inoculated and un-inoculated plants under stress were homogenized in ice cold potassium buffer (10ml, pH 7.0) keeping in an ice bath. Homogenized mixture was centrifuged at 12,000 rpm, for 20 min at 4°C. Each supernatant was stored at 4°C and used for the determination of various antioxidant enzymes [49]. Superoxide dismutase (SOD) activity was estimated by mixing leaf supernatant with nitro blue tetra -zolium (NBT) and photoreduction was done by placing (25 minutes) under lamp below 15 W [50]. After illumination absorbance was checked at 560 nm, NBT without supernatant was used as calibration curve, enzyme activity was denoted by units of mg/protein. Catalase (CAT) content was estimated by observing the reduction of H_2_O_2_ in reaction mixture of enzyme. A 6 mM of H_2_O_2_ were added to leaf extract supernatant, after 2 minutes incubation optical density for each treatment was recorded at 240 nm. Per-oxidase (POX) was analysed by monitoring the change at 420 nm in absorbance of guaiacol for 2 minutes due to its oxidation. The reaction mixture contained enzyme extract along with potassium phosphate buffer (50mM) and 1% H_2_O_2_ (0.1mM), 1 ml EDTA (0.1mM). Chance and Maehly method [51] was adapted to estimate catalase (CAT) and peroxidase (POD) contents. Yield parameters were recorded as number of pods/plant, pod length, number of grains/pod (taken randomly three replicates), weight of 100 seeds, weight of 100 seed straws and total yield (gm) for ten plants taken randomly.

### Statistical analysis

The data of all trials was statistically analyzed by using software IBM SPSS Statistics Ver. 22. The pot experiments were repeated thrice-using randomized block design (RCBD), while split-plot RCBD design was applied in the field trial. The data was expressed as the means of three or two replicates ± standard error of the replicates of every trial and were interpreted by analysis of variance (ANOVA one-way) followed by Duncan’s multiple range test (DMR) at the significance level of 0.05.

## Results

Five strains of rhizobacteria selected “S1 Fig”, were isolated from rhizospheres of four wild (*Ziziphus jujube*, *Melia azedarach*, *Calotropis procera* and *Cnicus arvensis*) and one from cultivated plant (*Mangifera indica*) from different cities of Punjab (Khanewal, Mian chanoo, Sheikhupura, Lahore) and were given the number M1, M4, M7, M11 and M15 (Later, given accession numbers MK513745, MK513746, MK513747, MK513748, MK513749 according to NCBI Genbank). Soil associated with them in analysis performed, was mostly blocky, sub angular or blocky loam. Soil pH ranged from 8 to 8.9 and E.C was between the range of 2.87 to 10.45 mS/cm. Phosphorus content was 4.1 to 5.9 ppm while potassium content ranged between 160 to 192 ppm “S1 Table”.

### Isolation, purification, characterization of rhizobacteria

Colonies obtained from five isolated strains after purification, were circular and greenish except M15 which had off white color. The colony margins of all 5 strains were entire and elevations were flat for MK513745, MK513748 and MK513749 while MK513746 and MK513747 were of raised elevation. The strain MK513745, MK513746, MK513747 and MK513749 had grape like odor while MK513748 had tortilla like smell. All strains were spore producing.

All isolated strains were gram negative rods and had the ability to oxidize carbohydrate. All strains were Vogus proskaur positive having reddish ring at the top of broth. All five were able to produce chitinase, catalase, and protease enzyme. Moreover, these were capable of swimming and swarming abilities. The strain MK513746 and MK513747 had swimming zones of 7.4 cm while MK513745, MK513748 and MK513749 produced 8, 8.4 and 8.5 cm, respectively. The strain MK513745 produced 6.3 cm swarming zone while MK513746, MK513747, MK513748 and MK513749 showed swarming zones of 7.1, 7.4, 7.7 and 8.06 cm, accordingly. The strain MK513749 had maximum swimming and swarming activities (8.5 and 8.06 cm respectively) “S2 Table”.

### Drought tolerance assay

All isolated strains were grown in broth amended with different concentrations of PEG 6000 to create stress levels of 0%, 5%, 10%, 15% and 20%. Isolates with high optical density after 3 days growth period were selected as drought tolerant strains. After 3 days of incubation broth for all stress levels were centrifuged and supernatants were used to detect optical densities. All five strains were drought tolerant and show growth up to 20% PEG stress, having optical densities more than 0.5.

### Evaluation of PGP characteristics

**Fig 1:**
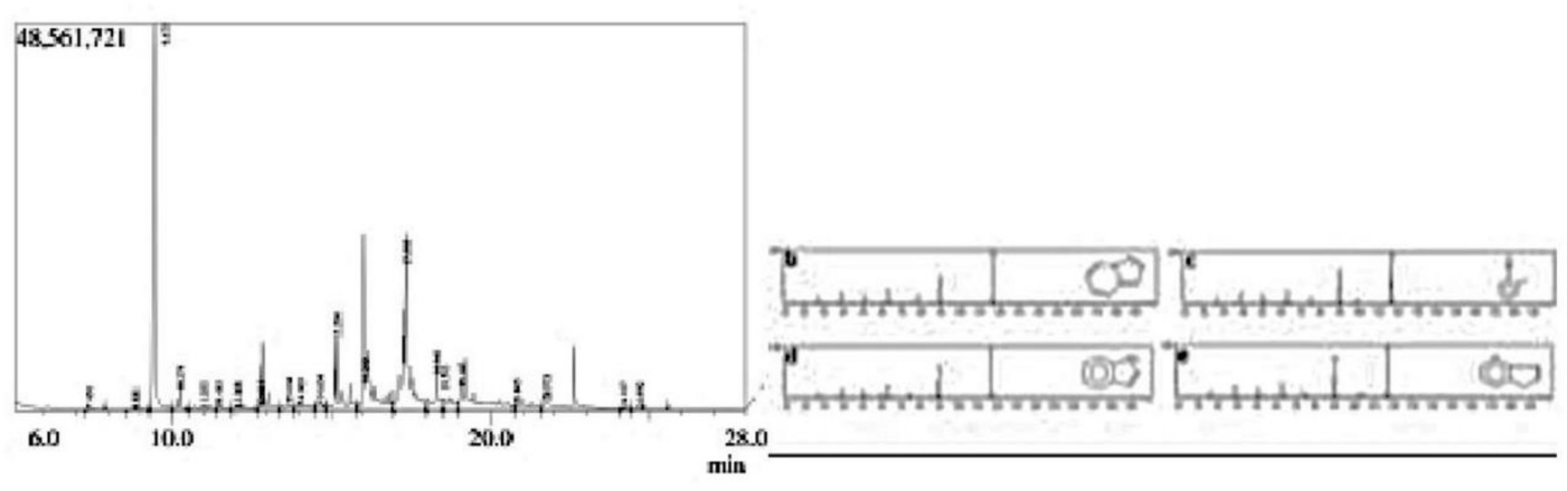
(a) Electrogram of MK-513749 auxin metabolites showing abundant peak (9.475 min); fragmentation of daughter ions in to (b) indolizine; (c) 2-methyle benzonitrile; (d) indole ring; (d) pyrin dine in GCMS analysis.

Capability of auxin production “S2 Fig” of selected strains was between values of 64 to 116 μgml^-1^ (by MK513746 and MK513745). Based upon auxin production abilities Strain MK513746 showed low auxin production (64 μgml^-1^), strain MK513747 and strain MK513748 (96 and 93 μgml^-1^) produced auxin more than that of strain MK513746 while MK513745 and MK513749 produced high concentrations of auxin in terms of 116 μgml^-1^ and 107 μgml^-1^ “S2 Table”. Spectra obtained by GCMS analysis showed 12 different indole compounds produced by *Pseudomonas aeruginosa* (MK513749) between 10 to 20 minutes time of retention (Table 1, Fig 1) when fragmented into daughter ions. Three major peaks were considered peak 3 having retention time 9.47 and base peak was 117.10 minutes. Indole was with 88% mass peak having formula C_8_H_7_N with molecular weight 117 indicated as a ring. Other daughter ions were cyclo penta pyridine, benzonitrile, indolizine and pyrocolin. In 13^th^ peak, whose retention time was 16.03 minutes having mass peak 153 and base peak 71.05, when fragmented. The main peak was fragmented into trybutyrin, Butanoic acid, butyril triglyceride, ethylene glycol butyrate with molecular formula C_15_H_26_O_6_ molecular weight 302 and 2-methyl Propanoic acid with C_8_H_14_O_3_ molecular weight 158. The 14^th^ peak fragmented in at 17.38 retention time into benzene dicarboxylic acid with molecular formula C_30_H_50_O_4_ and molecular weight 158. Other daughter ions were phthalic acid and hexa decanoic acid.

**Table 1:** Indole compounds identified in secondary metabolites of *Pseudomonas aeruginosa* MK 513749 in GCMS.

Compound	Retention time	Mol. Formula	Mol. Weight	Mass peak	Base peak
Cyclopenta Pyrindine	9.48	C_8_H_7_N	117	88	117.10
Benzonitrile, 2-methyl	9.48	C_8_H_7_N	117	88	117.10
Indolizine	9.48	C_8_H_7_N	117	88	117.10
Pyrrolo pyridine	9.48	C_8_H_7_N	117	88	117.10
Indolizin	9.48	C_8_H_7_N	117	88	117.10
Pyrrocolin	9.48	C_8_H_7_N	117	88	117.10
Pyrrocoline	9.48	C_8_H_7_N	117	88	117.10
Indole	9.48	C_8_H_7_N	117	88	117.10
Tributyrin	16.03	C_15_H_26_O_6_	302	153	71.05
tri-Butyryl triglyceride	16.03	C_15_H_26_O_6_	302	153	71.05
Glycerin tributyrate	16.03	C_15_H_26_O_6_	302	153	71.05
Ethylene glycol di-n-butyrate	16.03	C_10_ H_18_O_4_	202	153	71.05
Butanoic acid	16.03	C_8_H_14_O_3_	158	153	71.05
Propanoic acid	16.03	C_8_H_14_O_3_	158	153	71.05
2-methyl-anhydride	16.03	C_8_H_14_O_3_	158	153	71.05
1,2-Benzenedicarboxylic acid	17.38	C_30_H_50_O_4_	474	201	149.05
Hexadecanoic acid	17.38	C_16_H_32_O_2_	256	201	149.05
Phthalic acid	17.38	C_26_H_42_O_4_	418	201	149.05

Strain MK513745, MK513746 and MK513748 were recorded positive for phosphate solubilization, as they produced hallow zones around their colonies. Strain’s capabilities were quantified after 7–14 days of incubation in NBRIP medium plates. After incubation strain MK513746 had S.I. 2.6 mm, while strain MK513748 showed maximum S.I. (3.3 mm) “Table 2”. From standard curve, concentration of Phosphorus was calculated for each strain. Highest concentration was recorded in MK513748 which was 95 μgml^-1^. The strain MK513745, MK513746 and MK513748 were positive for N. fixation, producing colorless zones around their colonies. Activity Index of strain MK513745 was maximum (5 mm) followed by that of MK513748 and MK513746 (4 mm) after 7 days of incubation. In quantification method (Barton’s test) maximum Nitrogen concentration was 0.19 μgml^-1^ of strain MK513745 “Table 2”. All five strains showed siderophore producing activity, Blue colored CAS agar plates turned orange to yellow near colonies indicating production of siderophore. All strains were quantified by siderophore shuttle assay. Percent siderophore units ranged from 2.6% to 2.8%. Maximum production was recorded in strain MK513748, strain MK 513749 and strain MK 513747 “Table 2”; “S3 Fig”. Two strains were positive for HCN production MK 513746 and MK 513747, yellow colored filter paper dipped in picrate solution, placed in lid during incubation, turned brown for these strains. All selected strains were ACC deaminase production positive “Table 2” as they produced white colonies on D.F medium (Dworkin and Foster).

**Table 2:** PGP characterization of selected strains of *Pseudomonas aeruginosa*.

Parameters	Selected strains of Pseudomonas				
	MK513745	MK513746	MK513747	MK513748	MK513749
N fixation (SAI)	5±0.50^a^	4±0.16^c^	-	4±0.10^b^	3±0.01^d^
N. concentration (μg/ml)	0.19±0.01^b^	0.17±0.01^d^	-	0.18±0.01^c^	0.45±0.01^a^
P solubilization (S.I)	2.8±0.01^b^	2.6±0.11^c^	-	3.3±0.05^a^	-
P. Concentration (μg/ml)	91±0.13^b^	73±0.08^c^	^-^	95±0.13^a^	-
S.S. assay(% S. units)	2.6±0.05^d^	2.7±0.05^c^	2.8±0.05^b^	2.8±0.050^a^	2.8±0.07^b^
HCN production	-	+	+	-	-
ACC production	+	+	+	+	+

### Gene sequencing and phylogenetic identification of rhizobacteria

The nucleotide homology of five selected isolates was matched as 100% by database of NCBI (National Centre for Biological Information) with different strains of *Pseudomonas aeruginosa*. The gene sequences were submitted to NCBI GenBank and accession number was assigned as MK513745, MK513746, MK513747, MK513748 and MK513749 for M1, M4, M7, M11 and M15 strain respectively. MK513745 had 100% homology with *Pseudomonas aeruginosa* strain SSR 267, MK513746 had homology with *Pseudomonas aeruginosa* strain SB20, MK513747 had 100% homology with *Pseudomonas aeruginosa* strain PWC, MK513748 had 100% homology with *Pseudomonas aeruginosa* strain PS69 and MK513749 had 100% homology with *Pseudomonas aeruginosa* strain VITB SMSj5.

The phylogenetic tree was constructed with the help of nucleotide sequences according to their nearest neighboring strains showing their evolutionary relationship “S1 Fig”. All strains belong to Domain Bacteria, Phylum Protobacteria, Class Gammaprotobacteria, Order Pseudomonadales and Family Pseudomonadaceae.

### Growth promotion of *Vigna radiata*

#### Vegetative attributes

**Fig 2:**
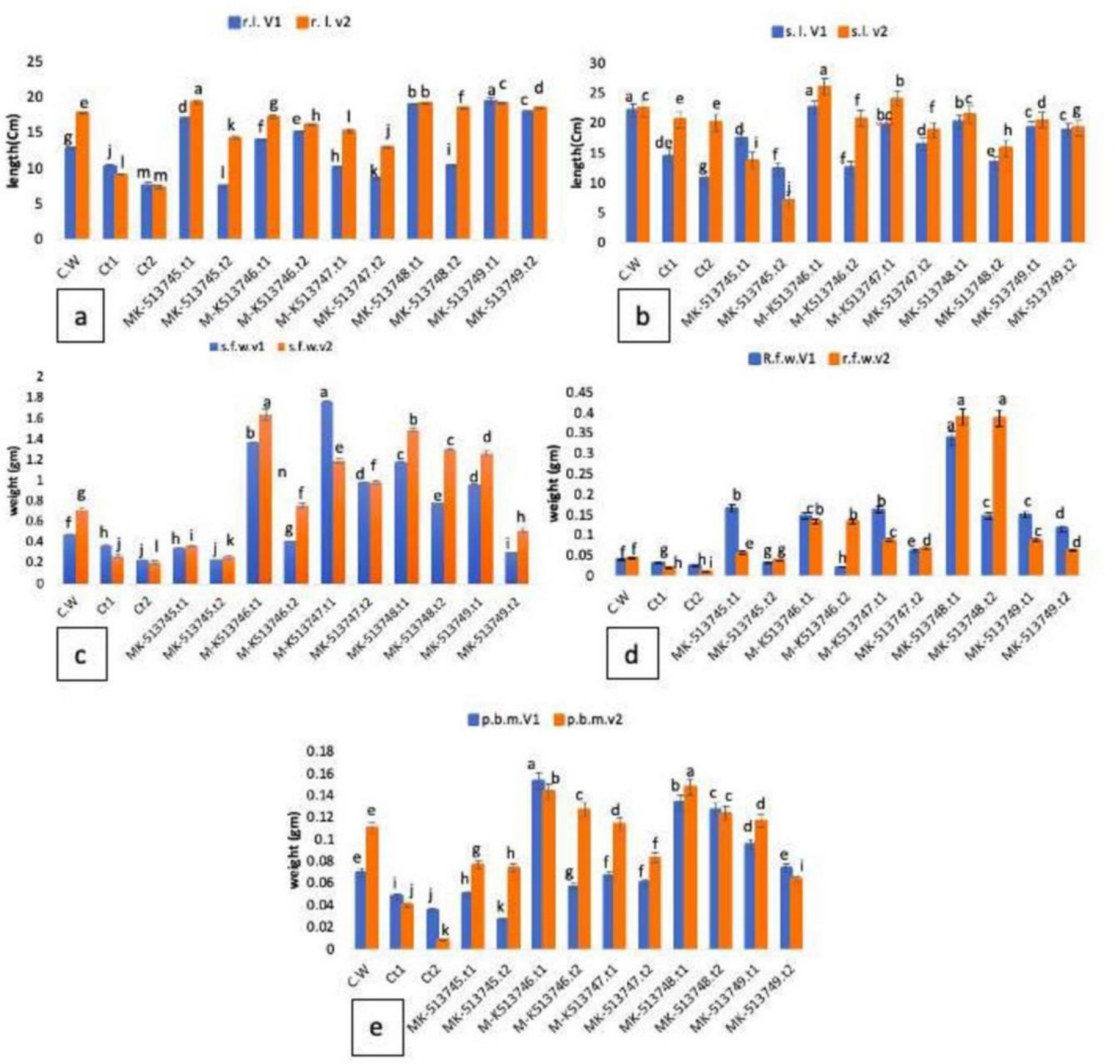
Effect of PGPR on the root length (a); shoot length (b); root fresh weight (c); shoot fresh weight (d) and plant dry biomass (e) of two different varieties of *Vigna radiata* grown under different drought conditions in lab trials. (t1 = 10% PEG and t2 = 20% PEG). The figure indicated traits varied significantly as a function of varieties (P<0.05), PEG (P<0.01) and strains (P<0.01).

In **lab trials**, root length decreased with increased concentration of PEG-6000 in all treatments (control plants, 10% and 20% stress) in both varieties. Plants inoculated with PGPR had more root lengths than stressed control plants. There was 52% increase with respect to control watered (C.W) and 90% increase as compare to control stressed (Ct1) in plants inoculated with PGPR MK513749 at 10% PEG stress in V1. Likewise, plants inoculated with strain MK513749 stimulated maximum root length at 20% PEG stress with 139% increase in root length (Fig 2A). In case of V2 same strain stimulated (MK513749) 8% increase with respect to control watered (C.W), 105% increase and 153% increase at 20% PEG stress as compared to uninoculated stressed (Ct1, Ct2 respectively) plants.

Maximum shoot length was observed in plants inoculated with strain MK513746 with % increases of 2.25, 16% as compared to C.W, 56%, 65% increases with respect to control at 10% PEG for V1 and V2 respectively whereas plants inoculated with strain MK513746, MK513747 stimulated shoot length with % increases of 74, 95% when compared with their respective control at 20% PEG stress in V1 and V2 respectively. Shoot heights of V2 were significantly better than V1 inoculated and un-inoculated plants (Fig 2B). Shoot fresh weights of plants with strain MK13746, MK513747, MK513748 and MK513749 inoculations were significantly higher at 10% PEG stress than at 20% PEG stress and all control (uninoculated) treatments (Fig 2C). Plants inoculated with strain MK513746 were stimulated maximum to accumulate weights with % increases of 389%, 531% in V1 and V2 at 10% PEG stress. The shoot fresh weights at 20% stress were less than those at 10% stress. Root fresh weights with MK513748 inoculations at 10% and 20% PEG stress of V1 and V2 were significantly higher (0.33 cm of V1 and 0.39 cm of V2) than control stressed (Ct1, Ct2) and control watered (CW) plants as well (Fig 2D). Performance of V2 was significantly better than V1 for these parameters. Plant bio masses with inoculations of MK513746, MK513747, MK 513748 and MK513749 at 10% PEG stress and strain MK513748 at 20% PEG stress were significantly higher (P>0.05) than both controls, at 10% and 20% PEG stress respectively (Fig 2E). Plant biomass with MK513748 inoculation was enhanced with percent increase of 275% in V1, 250% with MK513746, MK513748 in V2 respectively at 20% PEG stress. After successful interaction in axenic conditions, trials were performed in natural conditions for plant microbe interaction.

In **pot experiment**, root lengths of both varieties (V1 and V2) in both stress levels (50% and 75% field capacities) were significantly increased with PGPR inoculations as compared to un-inoculated plants. Shoot and root lengths of all PGPR treated plants at 75% field capacity (f. c) were greater than those at 50% f. c in both varieties and control plants as well. Increased stress level reduced the shoot lengths. Moreover, V2 responded better in all stress levels with PGPR application than un-inoculated plants of *Vigna radiata* “S3 Table”.

#### Physiological attributes

**Chlorophyll content** increased with increasing drought stress in all treatments “Table 3”. Inoculations with strain MK513747, MK513748 and MK513749 stimulated significantly higher accumulation of chlorophyll content. Maximum accumulation was recorded by MK513748 with % increases of 208%, 161% in V1 at 75% and 50% field capacity respectively likewise, 708%, 429% increase in chlorophyll content was recorded by MK513747 inoculations at 75% and 50% field capacity in V2 respectively as compared to their respective controls. Plants inoculated with PGPR of V1 produced more chlorophyll content than V2 inoculated plants (1.42 μgml^-1^ in strain MK513748 at 50% f. c).

**Table 3:** Effect of PGPR on physiological parameters of V*igna radiata* under different stress levels in pot trials.

Varieties	Strains	Chlorophyll content (μgml^-1^)		Relative electrolytes leakage %		Relative water contents %	
		75% F.C	50% F.C	75% F.C	50% F.C	75% F.C	50% F.C
V1	**C.W**	0.67±0.01^d^	0.66±0.01^f^	24±0.04^d^	24±0.04^f^	69±0.03^f^	69±0.01^c^
	**C.S**	0.39±0.01^f^	0.57±0.01^g^	27±0.07^a^	34±0.28^a^	61±0.21^g^	50±0.15^g^
	**MK513745**	0.68±0.01^d^	0.72±0.01^d^	20±0.14^e^	25±0.01^d^	81±0.01^c^	71±0.55^d^
	**MK513746**	0.61±0.01^e^	0.70±0.01^e^	19±0.49^e^	25±0.14^d^	78±0.14^d^	68±0.09^e^
	**MK513747**	1.05±0.01^b^	1.12±0.01^b^	27±0.07^b^	33±0.49^b^	72±0.21^e^	65±0.07^f^
	**MK513748**	1.21±0.01^a^	1.42±0.01^a^	25±0.21^c^	30±0.07^c^	89±0.07^b^	78±0.21^b^
	**MK513749**	0.94±0.01^c^	1.05±0.01^c^	17±0.07^f^	24±0.21^e^	92±0.49^a^	83±0.19^a^
V2	**C.W**	1.02±0.01^b^	1.02±0.01^b^	12±0.07^b^	17±0.35^b^	73±0.06^e^	73±0.06^e^
	**C.S**	0.13±0.01^g^	0.22±0.01^g^	20 ±0.03^a^	20 ±0.03^a^	66±0.07^f^	53±0.40^f^
	**MK513745**	0.22±0.01^e^	0.34±0.01^e^	11±0.07^c^	16±0.07^d^	84±0.14^d^	74±0.16^d^
	**MK513746**	0.20±0.01^f^	0.29±0.01^f^	11±0.21^c^	15±0.07^d^	89±0.70^b^	78±0.14^b^
	**MK513747**	1.06±0.01^a^	1.11±0.01^a^	12±0.01^b^	16±0.21^c^	88±0.07^c^	75±0.07^c^
	**MK513748**	0.61±0.01^d^	0.63±0.01^d^	10±0.07^c^	15±0.07^d^	91±0.20^a^	81±0.07^a^
	**MK513749**	0.79±0.01^c^	0.88±0.01^c^	7.75±0.07^d^	13±0.07^e^	84±0.70^d^	74±0.01^d^

C.W = unstressed control; C. S = Drought stress control.

**Relative electrolyte leakage** increased significantly with the increase of water stress. Plants with PGPR application showed significantly less electrolyte leakage especially in V2 plants (Table 5). Plants inoculated with strain MK513749 exhibited % decreases in relative electrolyte leakage (REL) with 36%, 36% in V1 and V2 respectively at 75% F. C, while at 50% F.C there was 31%, 36% decreases in V1 and V2 respectively as compared to their respective control plants under stress.

**Relative water content** was decreased with increasing drought stress level. Plants inoculated with PGPR showed increased water content as compared to un-inoculated plants “Table 3” in both varieties. Strain MK513749 inoculations stimulated 51%, 68% increases in RWC at 75% F. C, 50% F. C in V1 as compared to respective control. Whereas, strain MK513748 increased RWC accumulation with 38%, 54% increases at 75% F. C, 50% F.C with respect to control. Overall plants of V2 performed significantly better with inoculations at both stress levels in case of RWC.

In **field experiment** shoot lengths of all plants inoculated with PGPR were significantly higher than un-inoculated plants (C. S). Shoot lengths of V2 plants were more as compared to V1 plants whether inoculated or not. There were 124%, 80% increases in shoot lengths were recorded in plants inoculated with PGPR strain MK513745 of V1 and with strain MK513749 of V2 respectively when compared with respective control stressed plants (C.S).

#### Enzyme activity

*Stress osmolytes and antioxidant enzymes*. **Proline** content is a drought stress marker in plant leaves and was increased with drought stress application in plants as compared to non-stressed plants of both varieties “Table 4”. It was observed to increase with 142%, 925% increases in control stressed uninoculated (C.S) over control watered (C.W) plants in V1 and V2. Inoculations with PGPR strains adjusted stressed plants to accumulate this osmolyte with % increases of 308, 244, 235, and 188% over C.W with strain MK513747, MK513745, MK513746 and MK513749 respectively in V1 and 161.11% in V2 by strain MK513747. **Superoxide dismutase** (SOD**)** content was increased as 23%, 23% (V1, V2) in C. S plant leaves as compared to C.W plant samples. Plants inoculated with PGPRs stimulated accumulation of SOD content with 19% increase with MK513748 in V1 and 23% increase with strain MK513746 in V2 when compared to control stressed (C.S) plants.

**Table 4:** Effect of IAA producing PGPR on enzymatic pool of *Vigna radiata* in field trials.

Varieties	Strains	Proline (μmol/g FW)	SOD (unit/g fresh weight)	CAT (μmole/mg protein/min)	APX (μmole/mg protein/min)	POX (μmole/mg protein/min)	MDA (μ Mole)	H_2_O_2_ (unit/g fresh weight)
V1	**C.W**	**0.59±0.00** ^**f**^	**89±0.21** ^**f**^	**82±0.36** ^**a**^	**0.67±0.01** ^**f**^	**0.91±0.01** ^**f**^	**29±0.7** ^**f**^	**19±0.2** ^**f**^
	**C.S**	**1.43±0.01** ^**e**^	**110±0.2** ^**e**^	**60±0.20** ^**e**^	**0.76±0.02l** ^**e**^	**1.74±0.04** ^**e**^	**67±0.3** ^**a**^	**32±2.4** ^**a**^
	**MK51375**	**2.0±0.01** ^**b**^	**112±0.2** ^**e**^	**61±0.15** ^**e**^	**0.88±0.01** ^**d**^	**1.86±0.06** ^**d**^	**31±0.1** ^**e**^	**32±0.0** ^**a**^
	**MK51376**	**1.9±0.01** ^**c**^	**120.67±0.5** ^**d**^	**70.33±0.15** ^**d**^	**0.89±0.01** ^**cd**^	**2.64±0.04** ^**c**^	**41±0.1** ^**d**^	**29±0.01** ^**b**^
	**MK51377**	**2.4±0.01** ^**a**^	**126±0.1** ^**c**^	**74±0.36** ^**c**^	**1±0.01** ^**bc**^	**3±0.02** ^**b**^	**45±0.1** ^**d**^	**27±0.01** ^**c**^
	**MK51378**	**2±0.01** ^**e**^	**131±0.0** ^**a**^	**75±0.25** ^**b**^	**1±0.01** ^**b**^	**3±0.03** ^**b**^	**47±0.1** ^**b**^	**27±0.01** ^**c**^
	**MK51379**	**1.7±0.01** ^**d**^	**129±0.2** ^**b**^	**75±0.21** ^**b**^	**1±0.01** ^**a**^	**4±0.04** ^**a**^	**45±0.1** ^**c**^	**25±0.1** ^**d**^
V2	**C.W**	**0.5±0.01** ^**f**^	**91±0.40** ^**g**^	**84±0.25** ^**a**^	**0.68±0.01** ^**f**^	**1.1±0.04s**	**31±0.2** ^**f**^	**21±0.7** ^**f**^
	**C.S**	**0.5±0.02** ^**e**^	**113±0.8** ^**f**^	**62±0.26** ^**f**^	**0.78±0.01** ^**e**^	**2±0.01** ^**ef**^	**73±0.1** ^**a**^	**32±0.6** ^**a**^
	**MK51375**	**0.7±0.04** ^**c**^	**114±0.5** ^**e**^	**63±0.10** ^**e**^	**0.89±0.01** ^**d**^	**2±0.04** ^**e**^	**32±0.5** ^**e**^	**32±0.1** ^**a**^
	**MK51376**	**0.6±0.01** ^**d**^	**122±0.1** ^**d**^	**71±0.59** ^**d**^	**0.92±0.01** ^**c**^	**2.8±0.04** ^**d**^	**41.92±0.7** ^**d**^	**28.02±0.2** ^**b**^
	**MK51377**	**1.4±0.01** ^**a**^	**127±0.5** ^**c**^	**73±0.76** ^**c**^	**0.95±0.01** ^**ab**^	**3.5±0.06** ^**b**^	**45.88±0.1** ^**c**^	**27.01±0.1** ^**c**^
	**MK51378**	**0.6±0.01** ^**cd**^	**132±0.0** ^**a**^	**77±0.15** ^**b**^	**0.96±0.01** ^**ab**^	**3.3±0.01** ^**c**^	**48.15±0.7** ^**b**^	**26.51±0.9** ^**d**^
	**MK51379**	**0.7±0.01** ^**b**^	**131±0.3** ^**b**^	**77±0.12** ^**b**^	**0.97±0.01** ^**a**^	**3.7±0.0 1** ^**a**^	**46.36±0.06** ^**c**^	**24.18±0.01** ^**e**^

**Catalase** (cat) content was observed to reduce with 26%, 25% decreases in control stressed (C.S) plants (V1, V2) as compared to watered control plant. Plant growth promoting rhizobacterial inoculations stimulated 2% increase with strain MK513745 of V1 7% with strain MK513746 of V2 plants.

The amount **of ascorbate peroxidase** (**APX)** increased with drought stress application, there was 13%, 15% increases were observed in control stressed (C.S) plants (V1 and V2) with respect to control watered (C.W) plants. The plants with inoculations of strain MK513748 showed maximum accumulation of enzyme with 23% increase of V1 over respective C.S plants. The value of **peroxidase (**POX) content increased with treatment of drought stress “Table 4” and was calculated with 91%, 68% (V1, V2) increases as compared to control watered C.W. Inoculations with PGPR strains boosted POX production 104% in V1 with MK513748 and 96% with MK51374 while with MK513749 in V2 with respect to C.S plants.

#### Lipid peroxidative enzymes

Lipid peroxidation in leaf tissues was calculated in form of **malondialdehyde** (MDA) and hydrogen peroxide content. There was a significant difference between malondialdehyde (MDA) contents of control watered (C.W) and control stressed (C.S) plants (29 and 67 mmol/g FW), drought stress increased malondialdehyde (MDA) content in leaves of stressed control plants with 134% increase over C.W plants. Inoculated PGPR exhibited up-to 54%, 56% decrease in MDA content of V1 and V2 respectively as compared to stressed plants and tried to reduce lipid peroxidation and maintain its content like in control watered (C.W) plants (near to 29 mmol/g FW) at 0.05 P level. **Hydrogen peroxide** values increased with drought stress implementation. In control stressed (C.S) un-inoculated plants the content was observed with 62%, 54% increases when compared with control watered (C.W) plant samples “Table 4”. Inoculations with drought tolerant strains stimulated 30% decrease in Hydrogen peroxide content with strain MK51379 inoculations in V1.

#### Yield attributes

Number of pods per plant produced, were significantly higher in plants inoculated with PGPR than Control in both varieties. An increase of 39 to 46% as compared to respective control watered (C.W) and with 158 to 172% increases as compared to control stressed (C.S) was recorded in V1. Inoculations with strain MK 513749 stimulated pod production with 54.49% and 112.35% increases when compared with respective control watered (C.W) and control stressed (C.S) plants in V2 “Table 5”.

**Table 5:** Effect of IAA producing plant growth promoting rhizobacteria on growth and yield parameter of two varieties of *Vigna radiata* in field trials.

Varieties	Strains	Shoot Length (cm)	No. of pods/Plant	No. of Grains/pod	100 seed Wt.(g)	100 seed straw Wt.(g)	Total Yield (g)
V1	**C. W**	20±0.06^e^	4±1.50^b^	8±0.28^f^	6±0.01^a^	2±0.01^cd^	56±0.01^b^
	**C. S**	16.33±0.88^f^	2.33±1.04^f^	5.67±0.28^g^	3.50±0.02^e^	0.98±0.01^f^	24±0.03^f^
	**MK513745**	37±0.88^a^	6±0.76^d^	8±0.28^e^	5±0.01^d^	2.3±0.01^a^	26±0.01^f^
	**MK513746**	32±0.33^bc^	6±0.76^e^	9±0.28^cd^	4±0.02^d^	1.6±0.01^e^	38±0.03^e^
	**MK513747**	34.33±0.67^ab^	6.33±1.52^cd^	9.00±0.50^d^	5.34±0.01^b^	1.9±0.01^d^	49±0.01^c^
	**MK513748**	25.00±1.15^d^	6.33±1.25^b^	10.33±1.15^b^	5.07±0.00^c^	2.1±0.02^c^	44±1.70^d^
	**MK513749**	31.67±1.20^c^	6.00±2.17^a^	12.33±0.28^a^	5.83±0.01^a^	2.2±0.02^b^	60±0.11^a^
V2	**C. W**	24.7±0.15^c^	3.6±0.50^b^	10±0.01^d^	5.04±0.03^c^	2.2±0.05^d^	27±0.01^f^
	**C. S**	18.5±0.05^e^	2.6±0.28^c^	7±0.28^e^	3.94±0.12^d^	1.8±0.09^f^	20±0.25^g^
	**MK513745**	28.6±0.31^b^	5.6±0.50^b^	11±0.76^b^	6±0.02^c^	2.1±0.01^de^	48±0.03^c^
	**MK513746**	27.6±0.21^b^	5.3±0.58^b^	12±0.01^a^	6±0.01^c^	2±0.01^e^	45±0.13^e^
	**MK513747**	23.8±0.44^c^	5.0±1.52^a^	11±0.57^b^	6±0.02^c^	2.3±0.01^c^	47±0.01^d^
	**MK513748**	22.6±0.67^d^	5.6±0.28^b^	10±0.28^c^	7±0.03^b^	2.7±0.01^b^	57±0.01^b^
	**MK513749**	33±0.33^a^	5.6±0.28^b^	12±0.86^a^	8±0.20^a^	3±0.01^a^	77±0.01^a^

C. W = Watered control, C. S = Drought stress control.

**Fig 3:**
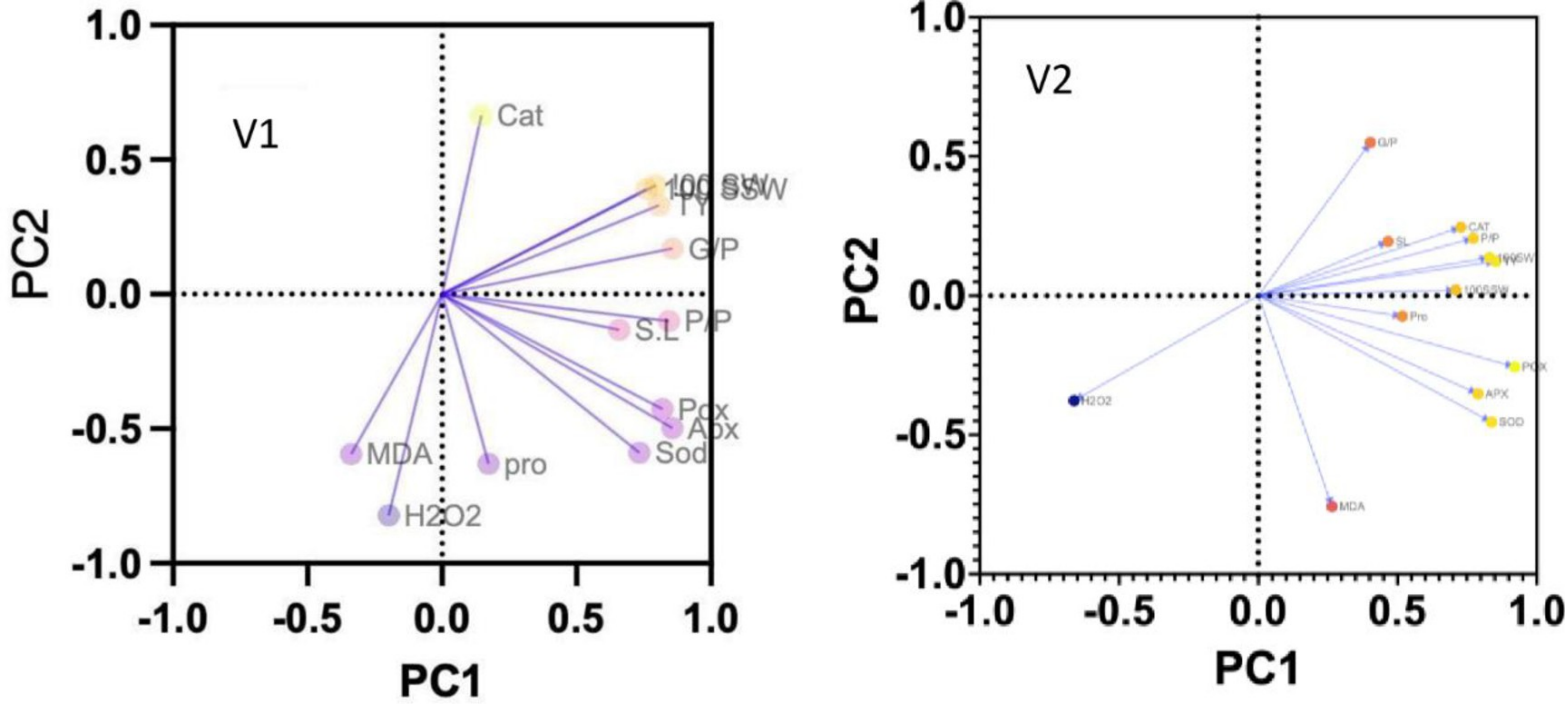
Biplot of PCA (principal component analysis) expressing different levels and relationship between enzyme productions, growth and yield parameter induction by drought tolerant strains in both varieties of *Vigna radiata* under water stress conditions. V1-AZRI 2006; V2-NM 2011.

Maximum number of grains/pod, 100 seed weight, 100 seed straw weight and total yield (ten plants) were recorded in plants inoculated with strain MK513749 in both varieties. Number of grains/pod was increased with PGPR application with % increases of 9% to 61% as compared to respective C.W and 47% to 117% increases as compared to respective C.S plants in V1 whereas, 7% to 20% and 46 to 64% increases were observed in V2 when compared with respective controls of watered and stressed plants. There was maximum increase in 100 seed weight of 67%, 95% increase in V1 and V2 respectively when compared with their respective C.S plants whereas, maximum yield was stimulated with strain MK513749 inoculation as 152%, 293% increases in V1 and V2 as compared to respective stressed control “Table 5”. The variety V2 responded better than V1 in growth, physiological and yield attributes (Fig 3).

## Discussion

The bacteria colonizing on plant root surfaces are nonpathogenic, improve plant growth and yield, directly or indirectly hence termed as PGPR [52]. Direct means of plant growth promotion are hormone production especially IAA; Phosphorus solubilization; Nitrogen fixation. Indirect means are production of other compounds (siderophores), metabolites (HCN) or enzymes (ACC deaminase production) which induce growth, suppress pathogens, and regularize production of other hormones. *Pseudomonas aeruginosa* is reported as plant growth promoter [53,54] and research widely in different crops. In the present study, five strains of Pseudomonas were isolated and characterized that were later explored for their capabilities as plant growth promoters in *vigna radiata* under drought stress.

Drought stress is an off-putting factor for plant growth and its production. In case of drought stress plants start to generate auxins as an adaptation. This effort slows down their other metabolic activities by reducing their dynamism along with improved root system and enhanced water uptake [55] this results, their hanged growth leading to death [56]. Inoculations plant growth rhizobacteria, capable of synthesizing auxins, preserve plants supplementary expanse of energy by contributing them with the required compounds [57]. This contributes to root, shoot growth, their architecture and increased biomass [58]. In the present study, thin layer chromatography (TLC) of methanolic extracts of rhizobacterial metabolites revealed the production of indole acetic acid (IAA) comparable to standard. Gas chromatography mass spectrometry results confirmed the presence of indole ring which is an important constituent of amino acid tryptophan, involved in production of auxin IAA in different pathways in PGP rhizobacteria [59]. The plants inoculated with auxin producing rhizobacteria exhibited more root and shoot growth as compared to un-inoculated plants (C.S) under different levels of drought stress in lab as well as in field conditions.

Phosphate solubilization and nitrogen fixing capability in PGPR mitigate drought stress in host plants by providing them with sufficient amounts of soluble phosphate, nitrates, iron and protection against pathogens [60]. The deficiency of phosphorus and Nitrogen directly causes poor stomatal conductance, less photosynthetic activity and reduces crop growth. Phosphate solubilizing rhizobacteria produce organic acids which improve P availability chemically and other growth substances hence stimulating plant growth [61]. A better root growth and their secretions for nitrogen, Phosphate solubilization when inoculated with PGPR are milestone for nutrient uptake and in result increase dry weight and plant yield [62].

The plant tissues increase ethylene production under abiotic stress, and an increased concentration of ethylene in plants can inhibit plant growth [63]. Plants inoculated with PGPR coupled ACC deaminase activity can alleviate plant growth inhibition owing to metabolic stress ethylene. The ACC deaminase produced by PGPR can reduce the ethylene concentration by cleaving the ethylene precursor ACC to α-ketobutyrate and ammonia; consequently, the plants maintain normal growth [64,65]. The Pseudomonas strains especially MK513749, MK513748 and MK513747 might be able to stimulate low ethylene concentration of mung bean plants by cleaving ACC under drought stress and hence increased plant growth in terms of root length, root and shoot fresh weights and plant dry biomass of all plants inoculated significantly higher than drought-stressed and non-stressed un-inoculated conditions (controls).This finding is supported by previous reports demonstrating increased resistance to abiotic stresses including drought [66–68].

Danish and Zafar-ul-Hye [69] reported that drought stress promotes poor stomatal conductance, less CO_2_ intake and restricted carboxylation hence leading to decreased rate of photosynthesis. Inoculations of PGPR with ACC activity increased photosynthetic activity under stressed conditions as compared to un-inoculated stress prone plants. These inoculations had a positive effect on the chlorophyll content of plants in both stress levels (75%, 50% F. C), hence improving the photosynthetic efficacy under water stress conditions [70–72]. The augmented amount of chlorophyll contents in plants inoculated with PGPR perhaps could be a source of amended plant growth under drought conditions. In consistent with this findings of [73] in canola, [74] in wheat, [75] in mung bean, [76] in soy bean are also reported.

Moreover abiotic stress damages cell membranes due to increased relative electrolyte leakage (REL) that is manifestation of stress level. Electrolyte leakage makes more porous to cell membrane, due to degradation of its lipid contents with the action of ethylene. These degraded lipids harm the cell integrity and ethylene then acts on chloroplast and activates its chlorophyllase (gene) hence damages the chlorophyll [72]. Inoculations with PGPR reduced cell membrane damage as compared to un-inoculated control plants [61,77,78]. Results of present study showed reduced electrolyte leakage in plants inoculated with growth promoting rhizobacteria as compared to un-inoculated stressed plants.

Application of PGPR inoculums improved relative water contents (RWC) in current study under drought stress and results resemble with the findings of [74,75] in wheat and Mung bean, respectively, with PGPR applications. These inoculations might reduce the drought inhibitory effects on root growth, aided in more effective root system formation to absorb more water from deeper soils [77].

Plants tend to normalize and shield themselves under drought by producing different osmolytes like proline which accumulate during abiotic stresses without disturbing their normal metabolism, helping them to attain stress tolerance and maintain growth [79]. Inoculations of PGPR in both varieties of mung bean induced stress tolerance by higher accumulations of proline. It is well documented that reason of reduced crop productivity under abiotic stresses is the production of ROS (reactive oxygen species) like hydrogen peroxide to which plant roots are very sensitive. In the presence of PGPR a full system of antioxidant activities is induced which scavenge ROS molecules [80]. During normal plant growth and development, a dynamic equilibrium between ROS and antioxidant defense contents has been observed in each of the aerobic cells. Under drought stress conditions, however, ROS content increased, disrupting the equilibrium in favor of oxidative reactions and causing oxidative stress. To prevent excessive ROS production, the plant activates antioxidant defense system comprised of enzymatic and non-enzymatic antioxidants [69,81] like superoxide dismutase (SOD), catalase (CAT), ascorbate peroxidase (APX), and malondialdehyde (MDA). In these antioxidants (enzymes that fight free radicals) superoxide dismutase (SOD) serves as the first line of defense against ROS-induced damage. It is a metalloenzyme (transcription factor) that is found in all aerobic organisms. Under stress, it catalyzes the reduction of reactive oxygen species to O_2_ and H_2_O_2_ [82]. Likewise, catalase is also an enzyme commonly found in all aerobic organisms, which catalyzes H_2_O_2_ into oxygen and water hence protects cells by these reactive oxygen species from oxidative damage [83]. PGPR inoculation was reported to enhance significantly the antioxidant enzymes (POD, SOD and CAT) activities and enhanced the accumulation of proline in mung bean under abiotic stress [84]. Hydrogen peroxide (H_2_O_2_) is regarded as an important redox molecule having remarkable intracellular stability (10^−3^ second’s half-life) due to its unique physical as well as chemical properties, and is capable of rapid and reversible oxidation of target proteins [85,86]. It can be transported across the cell membrane by aquaporins (carrier proteins) which results not only in long-distance oxidative damage contributing in regulation of cell signaling too [87]. More than 800 genes were reported to be detected in mature leaves of *Arabidopsis thaliana* by inoculations of *Piriformospora indica*, involved in reduction of ROS accumulation in roots, shoots and cell defense [88]. In present study, it was examined that inoculations with drought tolerant PGPRs prompted plants to produce the higher quantity of antioxidants (SOD, CAT, APX, POX, MDA) under water scarcity (drought) which may possibly be a cause of the less H_2_O_2_ accumulation in PGPR inoculated plants as compared to their respective controls in both varieties. However, lower accumulation of H_2_O_2_ in some PGPR treated plants (MK513747, MK513748, MK513749) indicated that this treatment combination might have developed a competent ROS quenching system at cellular level, which might help the plant to survive drought stress. Actually, PGPR possess enzyme ACC (1-aminocyclopropane-1-carboxylate-)-deaminase which is capable to inhibit the production of ethylene (plant retarding hormone) and promote the antioxidant defense system to improve tolerance against drought stresses in fabaceous crops [89,90].

Current studies also exhibited that based on root- shoot length, stages of pod formation and grain filling was more sensitive to water deficit as compared to flowering stage. In addition, results showed the PGPR with multitrait effects; mitigate drought stress and increased number of pods/plant, pod length and 100 seed weight in inoculated plants as compared to control plants. Sarwar et al [91] reported increased grain yield in mung bean by PGPR inoculation which enhanced P availability by chelation. Rhizobia and PGPR provide N for host and significantly increase number of grain/pod. This factor determines the grain yield as these strains (*Pseudomonas aeruginosa*) had ability to solubilize phosphorus by improving soil p content, physiological architecture of soil, organic matters and N content and aids their symbiosis with host plants [92].

Thus, inoculating plants with PGPR which have ability to produce IAA with additional characteristics of Nitrogen fixation, phosphate solubilization, Siderophore, HCN and ACC deaminase production might have aided them to cope with this abiotic stress. So, it is suggested that PGPR can be used to enhance crop productivity and reduce their dependence to chemical fertilizers.

## Conclusion

The findings of the present study are much useful to the agriculture sector for utilizing these potential beneficial bacteria as a bioinoculant which can successfully improve the stress tolerance and enhance the crop production under drought stress. Five bacterial strains belonging to the genus *Pseudomonas* have been isolated in this study. All five strains were drought tolerant and could produce IAA, ACC deaminase, siderophore and were capable of swimming and swarming activities. The strain MK513745, MK513746, MK513747, MK513748, MK513749 showed the possible ability to relief drought stress in *Vigna radiata* and improved grain yield significantly over control stressed plants in both varieties.

## Supporting information

S1 Fig Phylogeny of selected PGPR *Pseudomonas aeruginosa* strains evaluated in 16S rRNA Sequencing. (DOCX)

S2 Fig (a) Thin layer chromatography, pink bands showing presence of IAA (b) Salkowski test for IAA detection, yellowish coloration showing absence and pink presence of IAA in methanolic extracts of tryptophan dependent secondary growth of *Pseudomonas aeruginosa*.
(DOCX)

S3 Fig PGP characters and enzymatic activities, zones of (a) phosphorus solubilization (b) Nitrogen fixation (c) Gelatinase production (d) HCN production (e) Siderophore production.
(DOCX)

S1 Table The soil analysis of rhizospheric samples collected from different areas of Punjab. (DOCX)

S2 Table Biochemical and PGP characteristics of selected strains of Pseudomonas. (DOCX)

S3 Table Effect of PGPR on growth attributes of V*igna radiata* under different stress levels in pot trials. (DOCX)

S1 Graphical abstract (TIF)
