# Supplementary material for: Drought tolerance induction and growth promotion by indole acetic acid producing Pseudomonas aeruginosa in Vigna radiata
Source: PLoS One. 2022 Feb 4;17(2):e0262932. doi: 10.1371/journal.pone.0262932 (PMC8815908; doi:10.1371/journal.pone.0262932)
Supplement: S1 Fig — (DOCX) [file pone.0262932.s001.docx]

**S1 Figure Phylogeny of selected PGPR *Pseudomonas aeruginosa* strains evaluated in 16S rRNA**

**Sequencing**

**
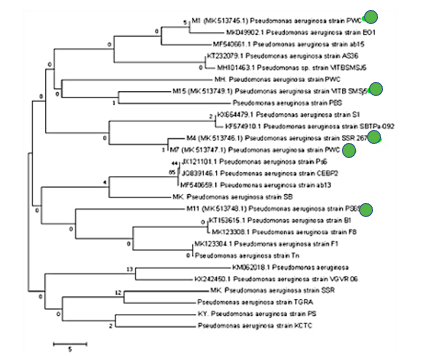
**
