# Supplementary material for: Drought tolerance induction and growth promotion by indole acetic acid producing Pseudomonas aeruginosa in Vigna radiata
Source: PLoS One. 2022 Feb 4;17(2):e0262932. doi: 10.1371/journal.pone.0262932 (PMC8815908; doi:10.1371/journal.pone.0262932)

**S2 Figure (a) Thin layer chromatography, pink bands showing presence of IAA (b) Salkowski test for IAA detection, yellowish coloration showing absence and pink presence of IAA in methanolic extracts of tryptophan dependent secondary growth of *Pseudomonas aeruginosa***


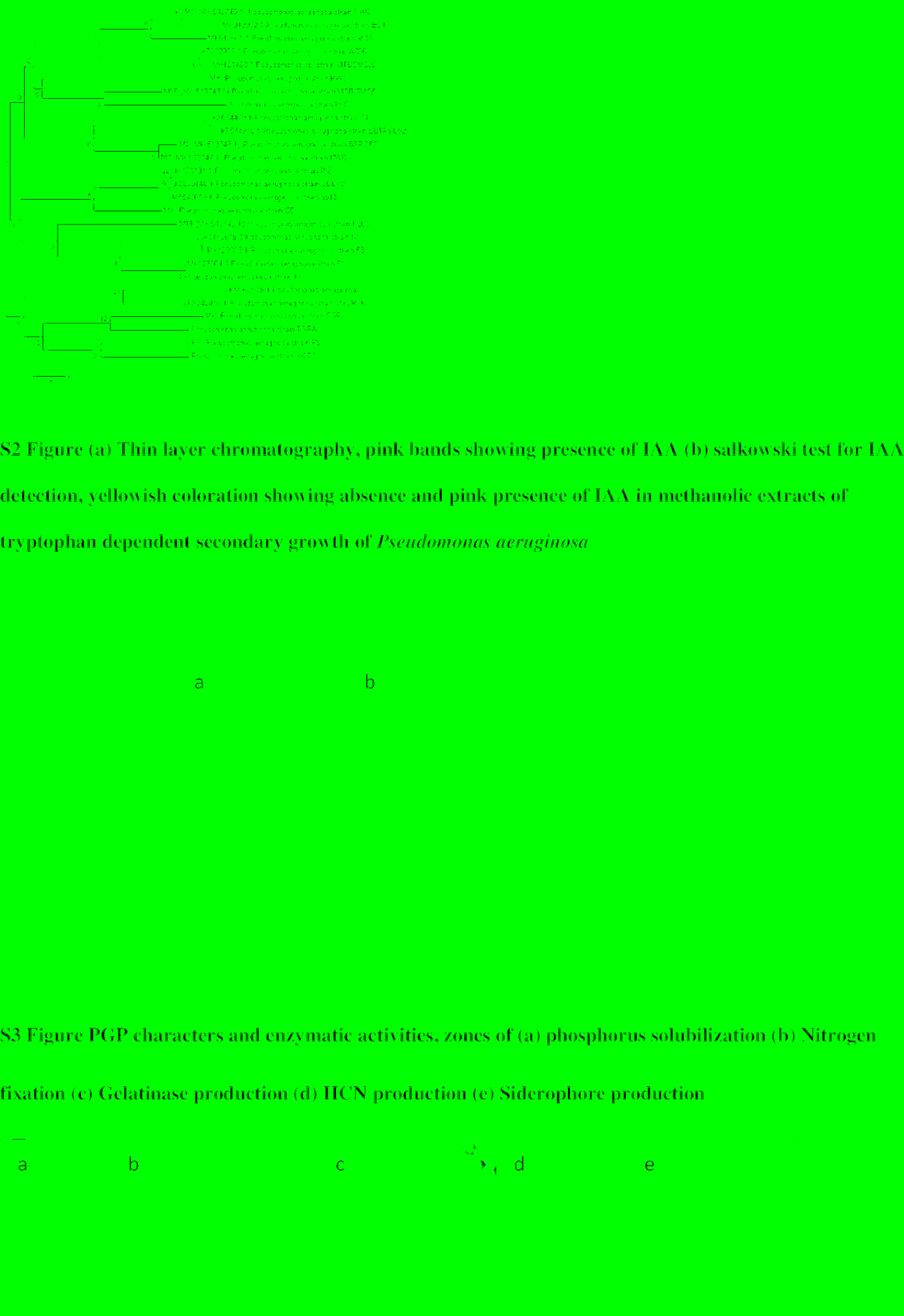

Supplement: S2 Fig — (a) Thin layer chromatography, pink bands showing presence of IAA (b) Salkowski test for IAA detection, yellowish coloration showing absence and pink presence of IAA in methanolic extracts of tryptophan dependent secondary growth of Pseudomonas aeruginosa. (DOCX) [file pone.0262932.s002.docx]
