# Supplementary material for: Drought tolerance induction and growth promotion by indole acetic acid producing Pseudomonas aeruginosa in Vigna radiata
Source: PLoS One. 2022 Feb 4;17(2):e0262932. doi: 10.1371/journal.pone.0262932 (PMC8815908; doi:10.1371/journal.pone.0262932)

**S3 Figure PGP characters and enzymatic activities, zones of (a) phosphorus solubilization (b) Nitrogen fixation (c) Gelatinase production (d) HCN production (e) Siderophore production**


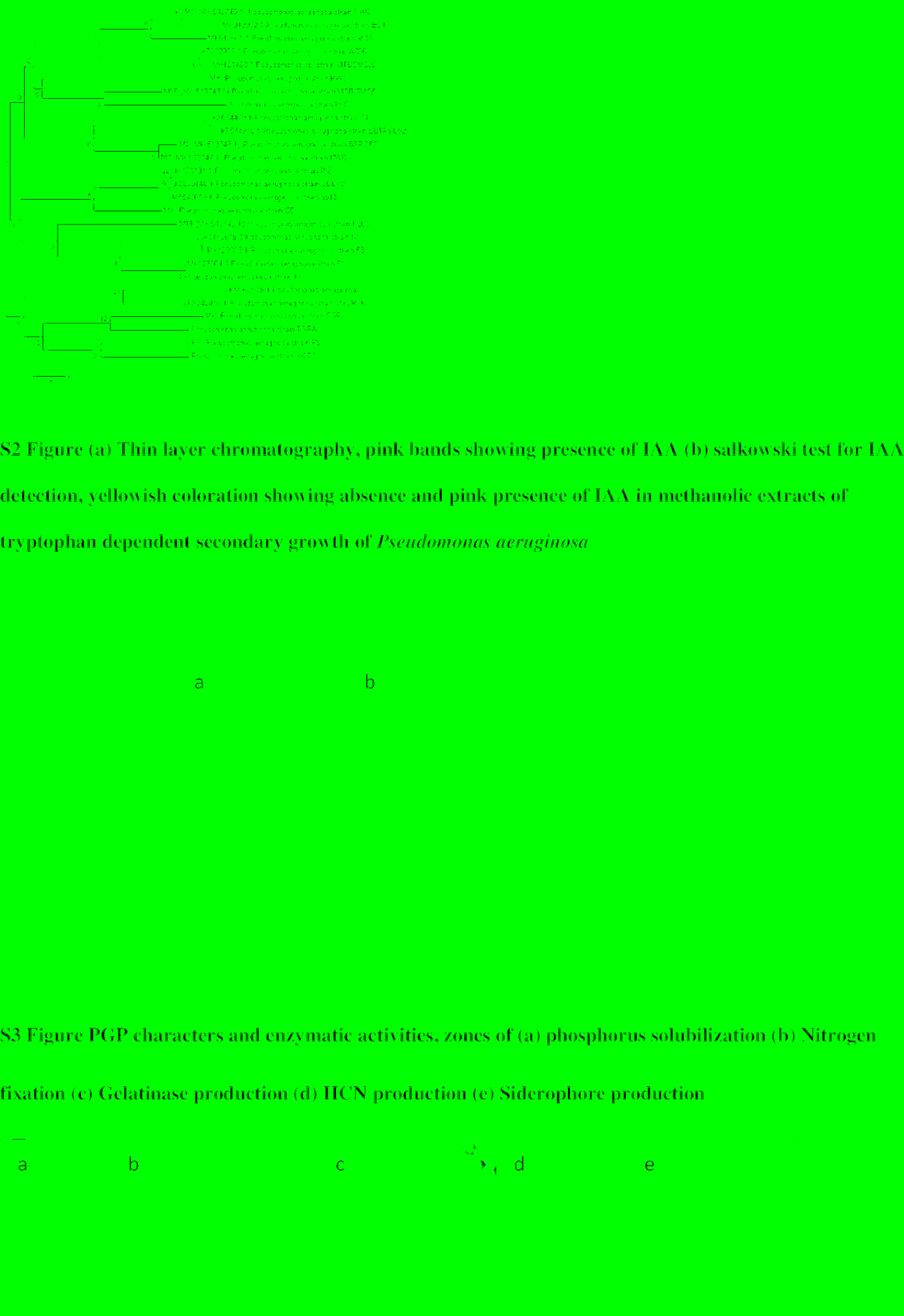

Supplement: S3 Fig — PGP characters and enzymatic activities, zones of (a) phosphorus solubilization (b) Nitrogen fixation (c) Gelatinase production (d) HCN production (e) Siderophore production. (DOCX) [file pone.0262932.s003.docx]
