# Supplementary material for: Drought tolerance induction and growth promotion by indole acetic acid producing Pseudomonas aeruginosa in Vigna radiata
Source: PLoS One. 2022 Feb 4;17(2):e0262932. doi: 10.1371/journal.pone.0262932 (PMC8815908; doi:10.1371/journal.pone.0262932)
Supplement: S1 Table — (DOCX) [file pone.0262932.s004.docx]

S1 **Table : The soil analysis of rhizospheric samples collected from different areas of Punjab.**

| Characteristics | Selected strains of Pseudomonas | | | | |
| --- | --- | --- | --- | --- | --- |
|  | MK513745 | MK513746 | MK513747 | MK513748 | MK513749 |
| Soil type (structure) | Blocky,  sub-angular  loam | Blocky, sub-angular loam | Blocky, loam | Blocky, loam | Blocky, loam |
| Soil pH | 8.1±0.025^d^ | 8.9±0.004^a^ | 8.0±0.028^e^ | 8.5±0.025^c^ | 8.7±0.023^b^ |
| O/ M % | 0.5±0.002^a^ | 0.4±0.002^b^ | 0.5±0.005^a^ | 0.3±0.005^c^ | 0.4±0.002^b^ |
| Soil EC mS/cm | 4.3±0.026^c^ | 2.8±0.010^e^ | 10.4±0.01^a^ | 3.5±0.020^d^ | 9.6±0.013^b^ |
| P content (ppm) | 4.3±0.025^c^ | 4.3±0.025^c^ | 4.1±0.016^d^ | 5.6±0.025^b^ | 5.9±0.007^a^ |
| K content (ppm) | 176±0.25^b^ | 172±0.25^c^ | 160±0.28^d^ | 176±0.25^b^ | 192±0.28^a^ |
