# Supplementary material for: Drought tolerance induction and growth promotion by indole acetic acid producing Pseudomonas aeruginosa in Vigna radiata
Source: PLoS One. 2022 Feb 4;17(2):e0262932. doi: 10.1371/journal.pone.0262932 (PMC8815908; doi:10.1371/journal.pone.0262932)
Supplement: S2 Table — (DOCX) [file pone.0262932.s005.docx]

**S2 Table : Biochemical and PGP characteristics of selected strains of *Pseudomonas aeruginosa* strains**

| **Parameters** | **Selected strains of Pseudomonas** | | | | |
| --- | --- | --- | --- | --- | --- |
|  | **MK513745** | **MK513746** | **MK513747** | **MK513748** | **MK513749** |
| **Gram staining** | - rod | - rod | - rod | - rod | - rod |
| **IAA Concentration (µg/ml)** | 115.71±0.13^a^ | 64.31±0.08^c^ | 96.07±0.07^c^ | 93.27 ±0.19^d^ | 107.58±0.26^b^ |
| **Swimming zones (cm)** | 8.0±0.25^c^ | 7.4±0.1^d^ | 7.4±0.2^d^ | 8.4±0.2^b^ | 8.5±0.25^a^ |
| **Swarming zones (cm)** | 6.3±0.25^e^ | 7.16±0.15^d^ | 7.4±0.2^c^ | 7.73±0.12^b^ | 8.06±0.22^a^ |
| **Catalase** | + | + | + | + | + |
| **Gelatinase** | + | + | - | - | + |
| **Amylase** | + | + | - | + | - |
| **Oxidase** | - | + | + | - | - |
| **Protease** | + | + | + | + | + |
| **Chitinase** | + | + | + | + | + |
| **HCN production** | - | + | + | - | - |
| **ACC production** | + | + | + | + | + |
| **Siderophore production** | + | + | + | + | + |
