# Supplementary material for: Drought tolerance induction and growth promotion by indole acetic acid producing Pseudomonas aeruginosa in Vigna radiata
Source: PLoS One. 2022 Feb 4;17(2):e0262932. doi: 10.1371/journal.pone.0262932 (PMC8815908; doi:10.1371/journal.pone.0262932)
Supplement: S3 Table — (DOCX) [file pone.0262932.s006.docx]

**S3 Table : Effect of PGPR on growth attributes of V*igna radiata* under different stress levels in pot trials.**

| Varieties | Strains | Shoot length | | Root length | | Plant fresh weight | | Plant dry weight | |
| --- | --- | --- | --- | --- | --- | --- | --- | --- | --- |
|  |  | 75% F.C | 50% F.C | 75% F.C | 50% F.C | 75% F.C | 50% F.C | 75% F.C | 50% F.C |
| V1 | C.S | 17.08±0.05^e^ | 14.13±0.07^f^ | 13.10±0.050^f^ | 8.40±0.050^e^ | 3.01±0.005^d^ | 2.50±0.025^e^ | 0.31±0.005^d^ | 0.21±0.002^d^ |
|  | MK513745 | 29.01±0.013^a^ | 22.50±0.250^b^ | 18.50±0.100^b^ | 8.01±0.002^e^ | 4.60±0.050^a^ | 3.03±0.020^c^ | 0.44±0.002^a^ | 0.29±0.005^b^ |
|  | MK513746 | 25.11±0.082^c^ | 16.74±0.190^d^ | 16.17±0.070^c^ | 15.03±0.018^b^ | 4.06±0.016^b^ | 3.17±0.023^b^ | 0.40±0.005^b^ | 0.29±0.002^b^ |
|  | MK513747 | 27.17±0.104^b^ | 18.00±0.190^c^ | 13.99±0.005^e^ | 13.03±0.076^c^ | 4.07±0.012^b^ | 3.07±0.010^c^ | 0.42±0.007^b^ | 0.29±0.005^b^ |
|  | MK513748 | 21.14±0.150^d^ | 15.00±0.100^e^ | 15.00±0.100^d^ | 12.01±0.005^d^ | 3.53±0.125^c^ | 2.60±0.050^d^ | 0.34±0.002^c^ | 0.26±0.004^c^ |
|  | MK513749 | 27.07±0.050^b^ | 24.98±0.014^a^ | 21.01±0.007^a^ | 19.20±0.260^a^ | 4.75±0.025^a^ | 4.21±0.008^a^ | 0.45±0.002^a^ | 0.40±0.005^a^ |
| V2 | C.S | 18.96±0.020^e^ | 17.99±0.005^e^ | 8.40±0.050^e^ | 11.90±0.080^e^ | 3.11±0.005^e^ | 2.64±0.018e | 0.31±0.001e | 0.25±0.002^f^ |
|  | MK513745 | 27.04±0.027^c^ | 25.00±0.057^b^ | 8.01±0.002^c^ | 20.01±0.007^c^ | 4.73±0.020^c^ | 4.02±0.014^c^ | 0.46±0.020^c^ | 0.39±0.002^d^ |
|  | MK513746 | 25.02±0.007^d^ | 21.02±0.014^d^ | 15.03±0.018^d^ | 18.47±0.104^d^ | 4.34±0.025^d^ | 3.60±0.002^d^ | 0.42±0.002^d^ | 0.35±0.003^e^ |
|  | MK513747 | 25.20±0.130^d^ | 23.04±0.090^c^ | 13.03±0.070^b^ | 21.10±0.086^b^ | 4.70±0.002^b^ | 4.23±0.020^b^ | 0.46±0.002a^b^ | 0.41±0.003^b^ |
|  | MK513748 | 28.27±0.120^a^ | 26.03±0.020^a^ | 12.01±0.070^a^ | 22.03±0.010^a^ | 4.92±0.028^a^ | 4.52±0.014^b^ | 0.47±0.010^a^ | 0.47±0.008^a^ |
|  | MK513749 | 27.70±0.302^b^ | 23.00±0.150^c^ | 19.20±0.264^c^ | 20.08±0.010^c^ | 4.78±0.015^b^ | 4.28±0.034^a^ | 0.46±0.002b^c^ | 0.40±0.003^c^ |
