# Supplementary figures and images for: Drought tolerance induction and growth promotion by indole acetic acid producing Pseudomonas aeruginosa in Vigna radiata
Source: PLoS One. 2022 Feb 4;17(2):e0262932. doi: 10.1371/journal.pone.0262932 (PMC8815908; doi:10.1371/journal.pone.0262932)

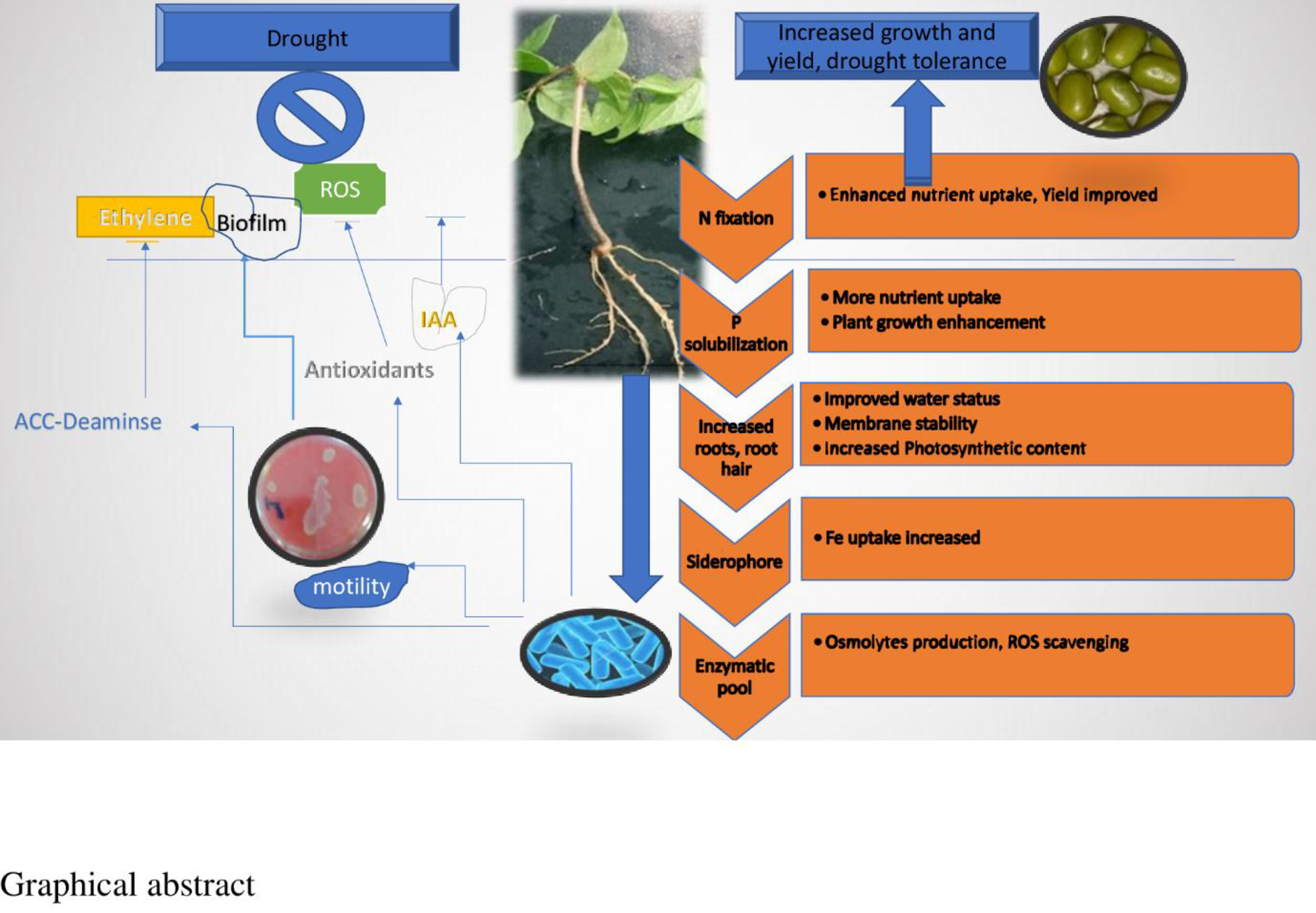

Supplement: S1 Graphical abstract — (TIF) [file pone.0262932.s007.tif]
